# *Wolbachia* dominance influences the *Culex quinquefasciatus* microbiota

**DOI:** 10.1038/s41598-023-46067-2

**Published:** 2023-11-03

**Authors:** Guillermo A. M. Flores, Rocio P. Lopez, Carolina S. Cerrudo, M. Alejandra Perotti, V. Fabiana Consolo, Corina M. Berón

**Affiliations:** 1 Instituto de Investigaciones en Biodiversidad y Biotecnología (INBIOTEC) - Consejo Nacional de Investigaciones Científicas y Técnicas (CONICET) and Fundación Para Investigaciones Biológicas Aplicadas (FIBA), Mar del Plata, Buenos Aires, Argentina; 2 Laboratorio de Ingeniería Genética y Biología Celular y Molecular (LIGBCM), Area Virosis de Insectos (AVI), Departamento Ciencia y Tecnología, Universidad Nacional de Quilmes and CONICET, Bernal, Argentina; 3 Ecology and Evolutionary Biology Section, School of Biological Sciences, University of Reading, Reading, UK

**Keywords:** Microbial communities, Applied microbiology, Computational biology and bioinformatics

## Abstract

Microorganisms present in mosquitoes and their interactions are key factors affecting insect development. Among them, *Wolbachia* is closely associated with the host and affects several fitness parameters. In this study, the bacterial and fungal microbiota from two laboratory *Culex quinquefasciatus* isolines (wild type and tetracycline-cured) were characterized by metagenome amplicon sequencing of the ITS2 and 16S rRNA genes at different developmental stages and feeding conditions. We identified 572 bacterial and 61 fungal OTUs. Both isolines presented variable bacterial communities and different trends in the distribution of diversity among the groups. The lowest bacterial richness was detected in sugar-fed adults of the cured isoline, whereas fungal richness was highly reduced in blood-fed mosquitoes. Beta diversity analysis indicated that isolines are an important factor in the differentiation of mosquito bacterial communities. Considering composition, *Penicillium* was the dominant fungal genus, whereas *Wolbachia* dominance was inversely related to that of Enterobacteria (mainly *Thorsellia* and *Serratia*). This study provides a more complete overview of the mosquito microbiome, emphasizing specific highly abundant components that should be considered in microorganism manipulation approaches to control vector-borne diseases.

## Introduction

Mosquitoes (Diptera: Culicidae) are considered the most significant medically important insects because of their impact on global public health, as they can vectorize a wide range of pathogens, including viruses and parasites, that affect millions of people each year^1^. Effective, cost-efficient, and safe control of these diseases requires the implementation of mosquito population control measures. However, the development of appropriate control strategies is a complex process that depends on multiple interrelated factors, including the specific mosquito species to be targeted, as well as biological, environmental, social, political, and economic considerations^2^. Microbiota-based approaches are currently being explored as novel mosquito biocontrol alternatives. Insect-associated microbiota include a variety of microorganisms, from free-living to parasites/pathogens and true symbionts, engaging in close and long-term biological interactions^3^. They can be part of the host environment and are hosted in specific structures within the body^4^. In mosquitoes, they occur at high loads in the midgut and may also colonize other organs or tissues, such as salivary glands and, in a few specific genera, the reproductive organs^5^. These microorganisms interact with each other to regulate development and population density^3^. On the other hand, mosquito microbiota interacts with their host, being important modulators of their phenotype, having a strong influence on multiple aspects of their life cycle, as well as affecting their nutrition, immunity, development, and even vector competence^5–7^. Different strategies have been explored for the use of native microbiota in the control of vector-borne diseases, including the selection of strains with better spreading or enhancing immune responses. In addition, genetic engineering has been proposed to produce effectors that interfere with pathogens^5^. Many microorganisms, such as the bacterial genera *Aeromonas*, *Asaia*, *Pseudomonas,* and *Serratia*; filamentous fungi, such as *Aspergillus* and *Penicillium*; and yeasts, such as *Candida* or *Pichia,* are commonly associated with mosquitoes. However, the mosquito mycobiome and its interaction with other microorganisms have been poorly studied compared with the bacteriome^7, 8^.

One of the most studied arthropod-associated bacteria, *Wolbachia pipientis* (Rickettsiales), has the ability to manipulate host reproduction. In mosquitoes, cytoplasmic incompatibility (CI) is induced, producing non-viable offspring in specific mating combinations. In general, CI allows *Wolbachia* to spread within insect populations through maternal transmission, effectively invading uninfected host populations and resulting in a selective reproductive advantage over uninfected females^9^. *Wolbachia* strains are capable of inducing variable effects on the phenotypes of different host mosquito species. In natural carriers, it has been observed to increase larval survival and longevity. In addition, reproductive fitness parameters, such as fertility, fecundity, and egg viability, can be positively or negatively affected^10–12^. In transinfected mosquito populations, this bacterium can reduce lifespan and alter locomotor and blood-feeding behavior^13^. Overall, vectorial competence may be affected by the regulation of the mosquito immune response, with mosquitoes becoming refractory to viral or parasitic infections^13, 14^.

In this work, the microbiota of the *Wolbachia* carrier *Culex quinquefasciatus* population maintained under laboratory conditions was analyzed and compared with a *Wolbachia*-cured mosquito isoline treated with tetracycline, which was allowed to recover its microbiota for many generations after treatment. The effect of the presence or absence of *Wolbachia* on fungal and bacterial diversity in both isolines throughout the development of the mosquito life cycle was studied through metagenomic analyses.

## Materials and methods

### Mosquito rearing and collection

*Culex quinquefasciatus* naturally infected with *Wolbachia* (*w*PipSJ strain) was originally obtained from natural breeding sites in San Juan Province, Argentina, and has been maintained under insectary conditions since 2016 (Biological Control Laboratory of the INBIOTEC-CONICET, FIBA). From the established mosquito line, wild type (wt), an antibiotic-treated isoline (-tet), was generated by exposing larvae to a tetracycline hydrochloride solution (0.1 mg/mL final concentration, Sigma, St Louis, MO; Cat. No. T33 83) during development until the pupal stage. Pupae were transferred to clean water without antibiotics and reared to adulthood. Adults were allowed to feed ad libitum with 10% sucrose containing 0.05 mg/mL tetracycline hydrochloride solution for three consecutive generations. After that, healthy insects were reared in dechlorinated water without antibiotics for three generations, and the entire process was repeated for three more generations. Finally, both isolines were reared in dechlorinated water at 24 ± 1 °C and a 12:12 h light/dark photoperiod. Larvae were fed on commercial fish food microorganisms free, and adults were provided with 20% (w/v) sucrose solution ad libitum; gravid females were also fed with mice blood. The presence of *Wolbachia* in both mosquito lines was checked through amplification of a *wsp* gene fragment using the primer combination *wsp*-81F and *wsp*-691R from gDNA, as previously described^15^. Isoline -tet has remained stable free of *Wolbachia*, without the addition of antibiotics*,* for more than 6 years, being checked monthly by PCR amplification of the *wsp* gene.

### Mosquito DNA extraction

A pool of 20 individuals from each of four groups was used for total genomic DNA extraction: (1) third instar larvae, (2) sucrose-fed males, (3) sucrose-fed females and (4) blood-fed females. Adults were first killed by freezing, and wings and legs were removed under a stereoscope. Dissected bodies were surface disinfected for 1 min with 70% ethanol, followed by 3 to 4 washes (1 min each) with sterile phosphate-buffered saline solution (PBS). Sucrose-fed adults were processed 48 h post emergence, and blood-fed females 96 h post emergence after blood digestion. For DNA extraction, DNeasy Blood & Tissue kit (QIAGEN) lysis buffer was added to insect samples in 1.5 mL tubes, frozen in liquid nitrogen and homogenized using a mechanical cell lyser and glass beads. Homogenates were incubated with 2 mg/mL proteinase K for 3 h for protein degradation and centrifuged at 12,000 rpm for 5 min. After that, the lysate was transferred to the kit column, following DNA extraction according to the manufacturer’s protocol^16^.

### Metagenome amplicon sequencing

Amplicon sequencing was performed using extracted gDNA previously quantified by the PicoGreen method through Victor 3 fluorometry (Invitrogen, Waltham, MA, USA) and quality checked by gel electrophoresis. From each mosquito group, libraries were constructed with primers targeting the nuclear ribosomal internal transcribed spacer (ITS2) and hypervariable regions V3–V4 of the 16S ribosomal DNA. Primer pairs used were ITS3 (5′-GCATCGATGAAGAACGCAGC-3′) and ITS4 (5′-TCCTCCGCTTATTGATATGC-3′)^17^ and 341F: (5′-CCTACGGGNGGCWGCAG-3′) and 805R: (5′-GACTACHVGGGTATCTAATCC-3′)^18^. Amplicons were sequenced on an Illumina MiSeq 300 bp PE platform in accordance with the manufacturer’s protocols. Library construction and amplicon sequencing were performed by Macrogen Inc. (Korea).

### Sequence analysis and taxonomic assignment

Paired-end reads were quality-checked by FastQC. Reads longer than 250 bp with an expected error < 1 were clustered into operational taxonomic units (OTUs) at 97% similarity using a combination of UPARSE pipeline^19^ and Mothur software, applying quality and chimera removal filters. Taxonomic assignment for fungi was performed on representative sequences from each OTU using massBLASTer and SH mappings of the UNITE database^20^. Bacterial OTUs were classified using Mothur against the SILVA database. Unclassified OTUs were taxonomically identified by BLAST searches against the GenBank standard database, nucleotide collection (nr/nt).

### Bioinformatic analysis

Using the raw reads for each sample, rarefaction curves were constructed in R, and bacterial and fungal filtered reads were used to estimate alpha-diversity indices in each community using vegan package 2.5^21^. For ITS2 samples, the reads of replicates were merged prior to diversity analysis. Relative abundance and heatmaps were graphed in GraphPad Prism 8.0.1. Principal component analysis (PCA) of all samples was performed with a combination of ggplot2 and ggfortify using the Galaxy web platform, usegalaxy.org^22^. Bray–Curtis dissimilarity indices for pairwise communities comparison were computed in the vegan package. Based on those values, a distance matrix was made to construct nonmetric dimensional scaling (NMDS) plots.

### Ethical approval

All the experiments were reviewed and approved by the Animal Experimental Committee at the Faculty of Exact and Natural Sciences, Mar del Plata University (Institutional Committee on Care and Use of Experimental Animals (CICUAL) N° 2555-04-14 and RD-2021-623). The animal experiment complies with the ARRIVE guidelines in strict accordance with National Health Service and Food Quality (SENASA) guidelines (Argentina), following the 2011 revised form of The Guide for the Care and Use of Laboratory Animals published by the U.S. National Institutes of Health.

## Results

Metagenomic sequencing from 16S V3–V4 and ITS2 ribosomal DNA regions revealed a total of 1,765,410 bacterial and 3,049,778 eukaryotic paired-end raw reads (Tables S1 and S2).

Rarefaction curves confirmed that the sequencing depth was sufficient to cover the bacterial diversity in all mosquito groups (Fig. S1). Eukaryotic ITS2 reads from all stages reached a plateau, indicating that the fungal community was well represented after two rounds of sequencing (Fig. S2).

### Bacterial alpha diversity

Five hundred and seventy-two (572) bacterial OTUs were clustered from the 16S rRNA amplicon reads. Richness values in larval samples were similar between wt and -tet isolines. In sugar-fed adults, tetracycline-treated richness decreased by at least half in comparison to wt. In blood-fed females, a contrary tendency was determined, displaying higher richness than the -tet line. In the wt, Shannon and InvSimpson indices values were highest in the early stage and decreased in the adult samples. In particular, blood-fed females showed over fivefold lower diversity than larvae. Similarly, the -tet line also presented a differential behavior: females displayed greater diversity than larvae, especially after blood-feeding (Table 1).

**Table 1:** Indices of richness and diversity in bacterial communities per *Culex quinquefasciatus* group in two isolines.

16S rRNA^a^	OTUs	Reads	Dominance	Margalef	Fisher_alpha	Shannon	InvSimpson
L	153	23,104	0.13	15.13	21.90	2.5	7.72
L-tet	171	22,280	0.56	16.98	25.20	1.38	1.79
M	192	30,000	0.44	18.53	27.44	1.47	2.30
M-tet	72	26,478	0.72	6.97	9.02	0.81	1.38
SF	176	44,164	0.85	16.36	23.32	0.62	1.17
SF-tet	73	31,917	0.26	6.94	8.92	1.65	3.87
BF	139	53,049	0.87	12.69	17.31	0.45	1.14
BF-tet	179	30,538	0.24	17.24	25.21	2.22	4.25

^a^*L* larvae, *M* sucrose-fed male, *SF* sucrose-fed females, *BF* blood-fed females. Tetracycline treated isoline is indicated with the suffix (-tet).

### Fungal alpha diversity

Sixty-one (61) different fungal OTUs resulted from the total samples, with great variation between them, from 2 OTUs in wt blood-fed females to 32 in larvae. For this line, richness decreased during development from larvae to adult stage, while -tet line larvae and sugar-fed adults did not show great differences. Remarkably, blood-fed females of both isolines showed a drastic reduction in the total mycobiota (2 and 3 OTUs) (Table 2). Shannon and InvSimpson indices computed for each sample revealed the highest diversity in -tet sugar-fed females, in contrast to its counterpart in the wt line presenting the lowest values of all samples.

**Table 2:** Summary of fungal community richness and alpha diversity per *Culex quinquefasciatus* group in two isolines.

ITS2^a^	OTUs	Reads	Dominance	Margalef	Fisher_alpha	Shannon	InvSimpson
L	32	9685	0.43	3.14	4.12	1.09	2.33
L-tet	18	34,629	0.61	1.53	1.83	0.70	1.65
M	19	4231	0.82	1.99	2.56	0.55	1.22
M-tet	21	8651	0.86	2.05	2.59	0.41	1.16
SF	13	1855	0.90	1.46	1.89	0.32	1.11
SF-tet	16	1164	0.31	1.94	2.62	1.69	3.22
BF	2	21	0.64	0.35	0.59	0.55	1.56
BF-tet	3	78	0.44	0.55	0.76	0.90	2.25

^a^*L* larvae, *M* sucrose-fed male, *SF* sucrose-fed females, *BF* blood-fed females. Tetracycline treated isoline is indicated with the suffix (-tet).

Considering all mosquito developmental stages, in wt, the greatest diversity was observed in larvae and decreased in adults independent of their food source. On the other hand, blood-fed samples showed very low richness but displayed higher diversity as a consequence of a more even distribution. -tet isoline sugar-fed females had the highest diversity, while wt had the lowest diversity among all mosquito stages.

### Bacterial communities composition in *Culex quinquefasciatus*

**Figure 1:**
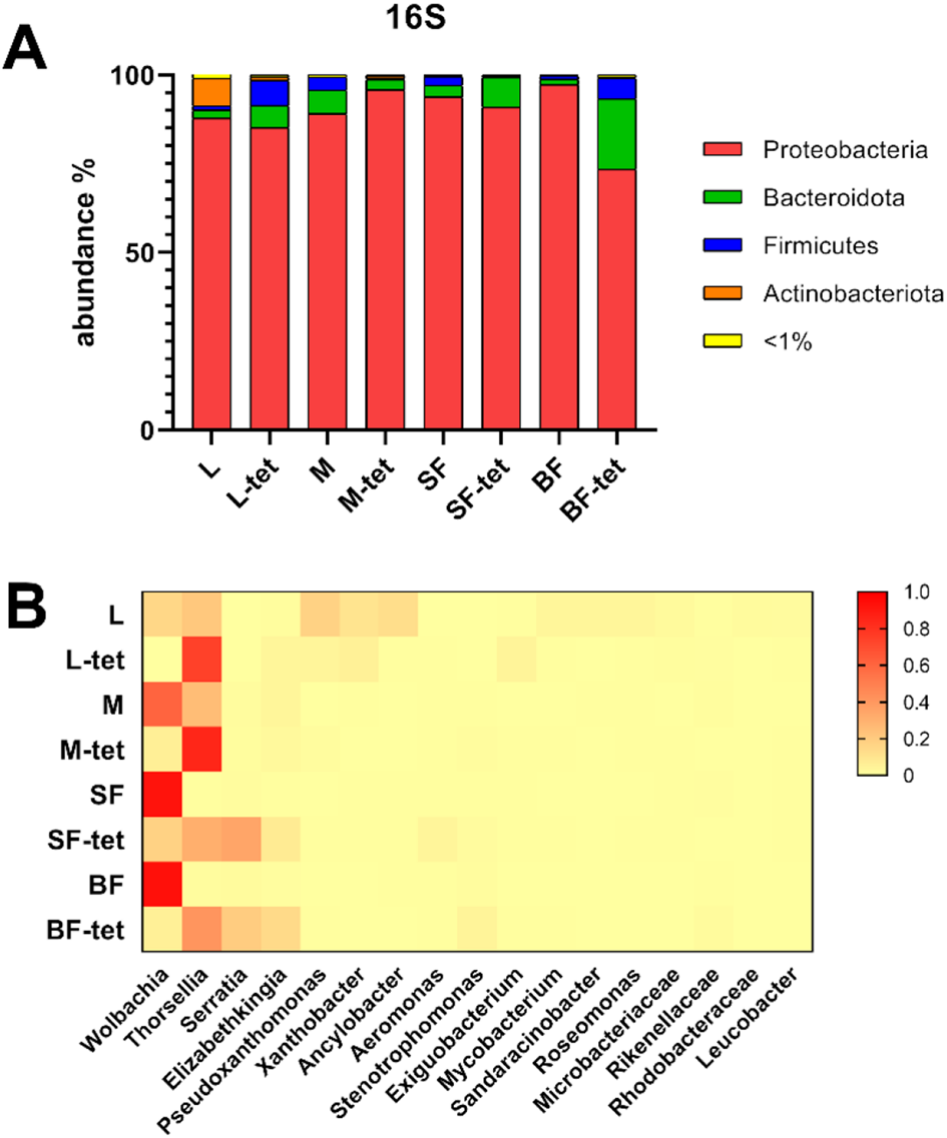
Bacterial relative abundance in wild-type and tetracycline-treated *Culex quinquefasciatus* isolines. (**A**) Bacterial community at the phylum level. (**B**) Heatmap of the relative abundance distribution of the most abundant bacteria in mosquito stages. Red color indicates higher relative abundance. *L* larvae, *M* sucrose-fed male, *SF* sucrose-fed females, *BF* blood-fed females. Tetracycline treated isoline is indicated with the suffix (-tet).

Bacterial communities were largely dominated by the phylum Proteobacteria, with a major relative abundance of more than 70% in all samples (Fig. 1A, Table S3). *Wolbachia* (Anaplasmataceae) had a high relative abundance in adults of the wt line (60.6–93.4%), although it represented 15% of the larval reads. Instead, in the -tet isoline, this OTU was absent in larvae, while in adults, it occurred in a lower proportion than in wt (5.6–17.4%). In all tetracycline-treated mosquito groups, the *wsp* gene was never detected by PCR amplification, while it was amplified in the wt groups (results not shown) (Fig. 1B, Table S3).

Another highly represented taxon was Thorselliaceae, with the highest values in larvae and males in wt (21.3 and 2.9%, respectively), even though this family was absent in females. In contrast, in the -tet isoline, Thorselliaceae was dominant in all mosquito stages. Remarkably, *Serratia* (Yersiniaceae) was the only genus present in both sucrose- and blood-fed females (20%). Xanthobacteraceae was prominent in wt larvae, with an abundance greater than 20%, distributed between *Ancylobacter* and *Xanthobacter*. Another remarkable OTU corresponded to *Elizabethkingia* (Weeksellaceae), which was more abundant in the -tet isoline, especially for females, reaching 14.2% in blood-fed and 7.61% in sugar-fed. Several bacterial genera were found to be considerably abundant in larvae samples, whereas they occurred at low frequency or were absent in adults.

### Fungal communities composition in *Culex quinquefasciatus*

**Figure 2:**
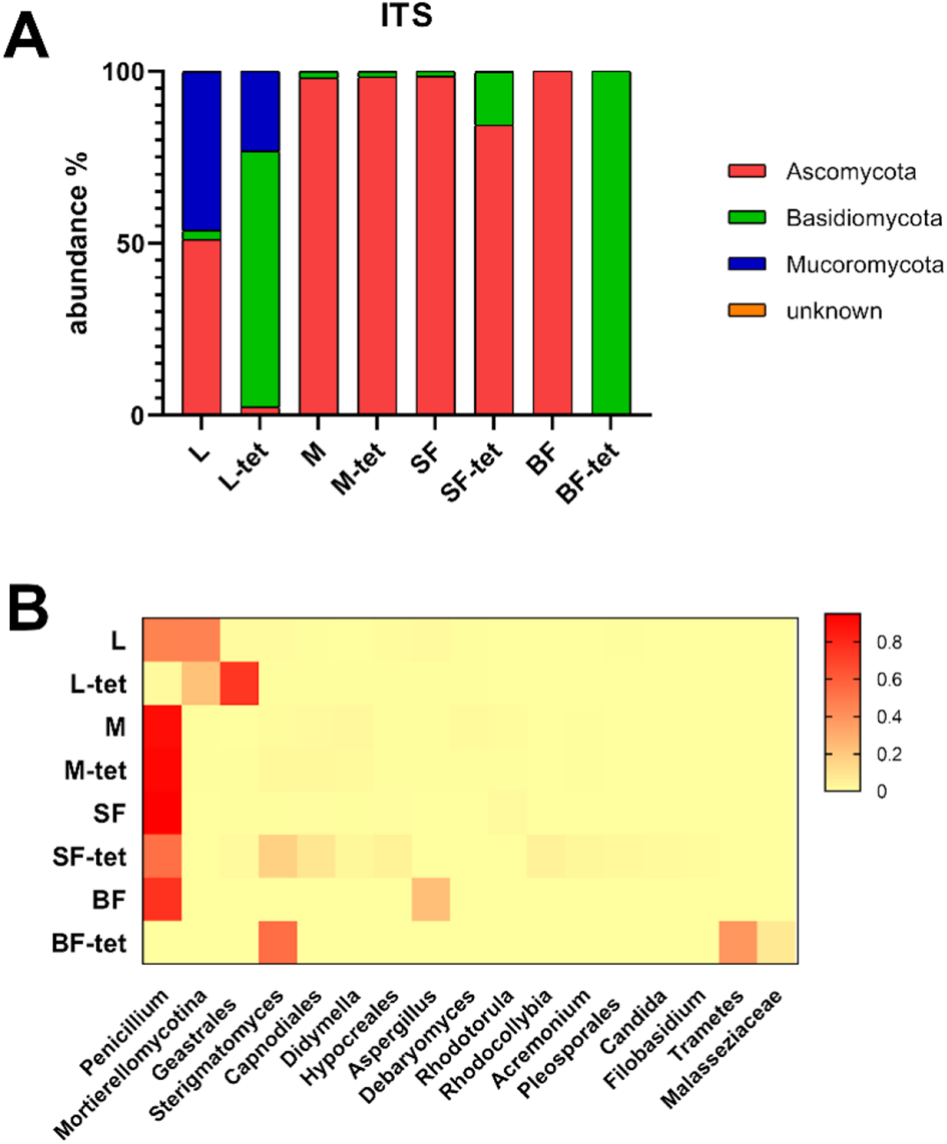
Fungal relative abundance in wild-type and tetracycline-treated *Culex quinquefasciatus* isolines. (**A**) Fungal community at the genus level. (**B**) Heatmap of the relative abundance distribution of the most abundant fungi in mosquito stages. Red color indicates higher relative abundance. *L* larvae, *M* sucrose-fed male, *SF* sucrose-fed females, *BF* blood-fed females. Tetracycline treated isoline is indicated with the suffix (-tet).

Samples in the wt isoline were dominated by Ascomycota (Fig. 2A). In particular, *Penicillium* had an abundance greater than 70% in all adult samples, although in larvae, one OTU of *Mortierellomycotina* (phylum Mucoromycota) showed equal relative abundance (Fig. 2B). Analysis of communities composition in the -tet isoline revealed a drastic reduction in *Penicillium* occurrence. Despite maintaining its dominance in sugar-fed mosquitoes, it was almost absent in larvae and blood-fed females. Particularly, in blood-fed females, Basidiomycota genera such as *Sterigmatomyces* and *Trametes* were identified, while the most abundant larval OTU was assigned to the basidiomycete order Geastrales. Two OTUs of filamentous fungi were more abundant in sugar-fed adults than in larvae samples, one belonging to the order Capnodiales and the other in the genus *Dydimella*. Both were absent in blood-fed females. Additionally, some genera, such as *Erysiphe* and *Rhodocollybia,* were unique to sugar-fed adults of the -tet isoline. Yeasts and yeast-like genera, such as *Sterigmatomyces*, *Debaryomyces*, *Rhodotorula*, *Candida* and *Malassezia,* were also identified (Fig. 2, Table S4).

### Beta diversity

**Figure 3:**
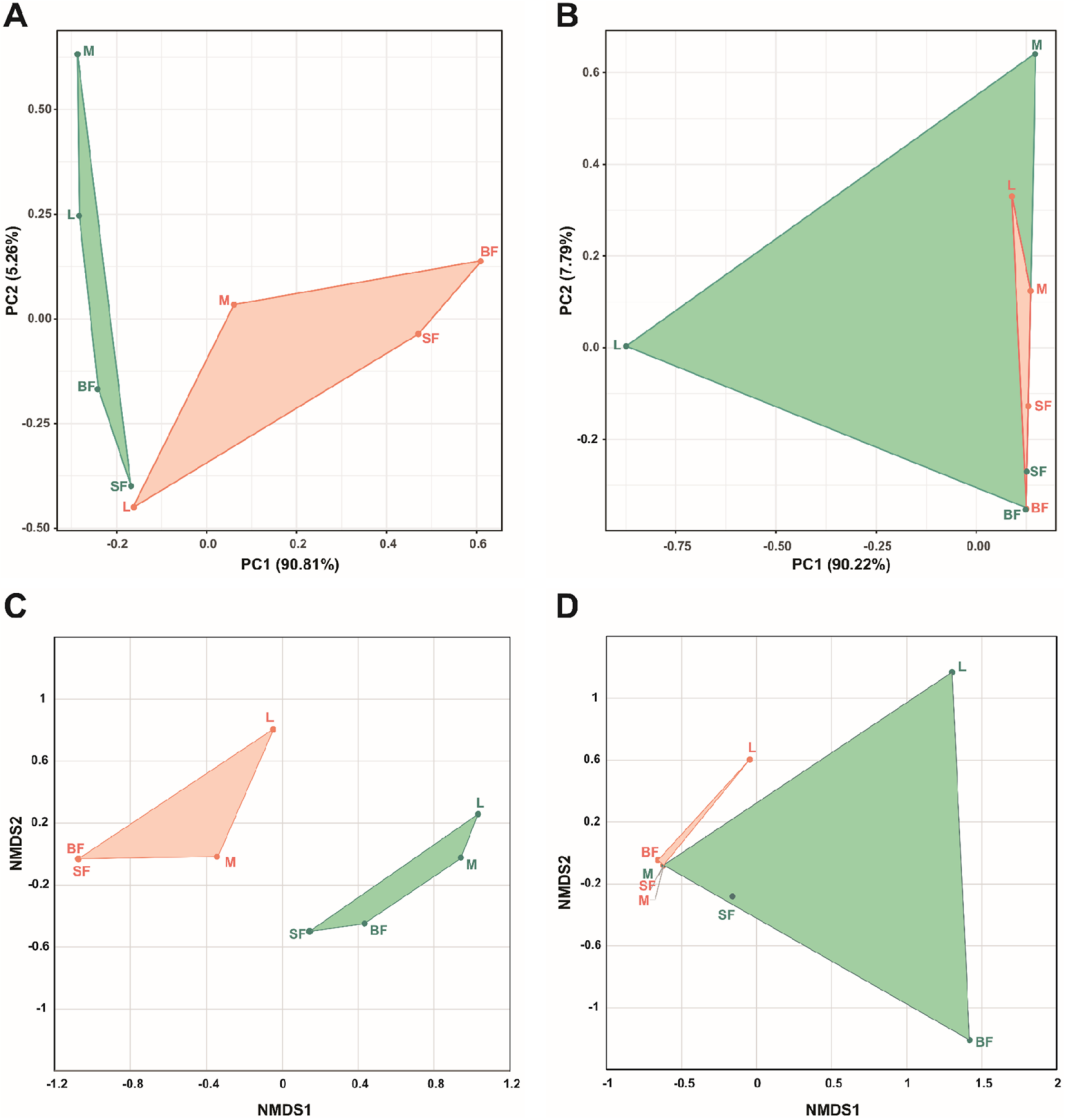
Principal component analysis (PCA) score plots and nonmetric multidimensional scale ordination (NMDS) plots of microorganism communities. Both analyses were performed for bacterial (**A**, **C**) and fungal (**B**, **D**) communities. In PCA score plots, based on OTU raw counts, X and Y axes show principal component 1 and principal component 2, which together explain 96.07% of the total variance (PC1: 90.81% and PC2: 5.26%) in 16S rRNA samples (**A**) and 98.01% of the total variance (PC1: 90.22% and PC2: 7.79%) in ITS2 samples (**B**). The results of the tetracycline-treated samples are represented in green, and the untreated samples are shown in red. *L* larvae, *M* sucrose-fed male, *SF* sucrose-fed females, *BF* blood-fed females.

The principal component analysis (PCA) plot for bacterial communities displayed both isolines as separate groups, with PC1 explaining that almost 91% of the variance was due to the differences between isolines (colored groups), while PC2 was associated with variance between stages (Fig. 3A). The Bray–Curtis-based NMDS showed a similar pattern, explaining the dissimilarities in the bacteriome between both populations as distant groups (Fig. 3C). In contrast, the PCA performed for fungal communities assigned most of the variation to PC1, in which samples were close-distanced. Only the wt larval sample was highly separated from the rest. NMDS showed an overlap in points representing samples from both isolines, supporting the PCA in which there was no separation of groups (Fig. 3B, D). In both microbial sets, larval samples were separated from the adults of their own isoline, indicating the presence of distinct and abundant OTUs. In addition, blood-fed female samples tended to have different communities from most samples in pairwise comparisons (Fig. 3, Tables S5 and S6).

## Discussion

In this work, bacterial and fungal microbiome from two laboratory *Cx*. *quinquefasciatus* isolines (wt and -tet) were compared throughout mosquito development, including changes in female feeding. Bacterial communities in the wt line presented higher Shannon diversity in larvae than adults, as has been demonstrated in a *Wolbachia* carrier *Ae. albopictus* population^23^. On the other hand, the -tet isoline showed a variable diversity distribution between developmental stages, detecting less diversity in larvae than in females. Meanwhile, in previous research, *Cx. tarsalis* naturally free of *Wolbachia* exhibited a similar diversity index among all stages^24^, and *Wolbachia*-free *An. atroparvus* population presented greater diversity in larvae than in females^25^. All these different examples highlight the diverse stage-specific interactions between microbes and their host.

In this study, the bacterial composition at the phylum level showed a dominance of Proteobacteria in all samples. In particular, OTUs such as *Thorsellia*, *Serratia, Aeromonas* and *Elizabethkingia* have been identified, some of which are commonly found in mosquitoes of the *Culex* genus^26–28^. The relative abundance data showed a different pattern between the most abundant genera when comparing the -tet and wt isolines. Samples with a high abundance of *Wolbachia* presented a reduced load of the enterobacteria *Thorsellia* and *Serratia*, and vice versa. As reported by Hegde et al.^26^, interaction network analysis based on metagenomic data from mosquitoes indicated that some bacteria could interact with each other, producing exclusion patterns in which the colonization of the microbiota by one OTU can inhibit the presence of another; finding that *Serratia* and *Aeromonas* would be co-excluded by *Wolbachia* in adult *Cx. quinquefasciatus* and *Ae. albopictus*. Additionally, an exclusion effect of the enterobacterium *Serratia* by *Cedecea* was experimentally demonstrated in *Cx. quinquefasciatus* and gnotobiotic *Ae. aegypti* systems^29^. Competition for ovary colonization by the acetobacterium *Asaia* has also been reported in natural *Wolbachia* carrier mosquito species^30^. Furthermore, in *Anopheles*, antibiotic treatment for *Asaia* reduction allows the vertical transmission of *Wolbachia*^31^. Interestingly, in *Cx. tarsalis* (naturally lacking *Wolbachia* endosymbiont) *Thorsellia* was present across all developmental stages in field-collected samples^24^. In this work, *Thorsellia* was detected in all stages of development, showing a considerable abundance increase in the -tet isoline. This would suggest that the pattern of dominant bacteria could be explained by an exclusion effect due to competition between *Wolbachia* and the enterobacteria.

Additionally, the influence of blood intake on the microbiome composition was also analyzed. It has been described that in the *Cx. quinquefasciatus* females dominated by *Wolbachia*, blood ingestion produced a drastic reduction in this genus in the midgut, accompanied by an increase in enterobacteria, indicating that *Wolbachia* would not participate in the digestion of blood^32^. Our data showed that in the wt isoline, the *Wolbachia* load remained at similar values, whereas the abundance of Enterobacteriaceae increased after blood ingestion. *Serratia* increased twice in blood-fed females than in sugar-fed females of the untreated isoline. However, the opposite trend was observed for the -tet isoline. Gaio et al.^33^ conducted in vivo experiments using antibiotic-treated *Ae. aegypti* and found that the microbiota actively participates in blood digestion. Furthermore, *Enterobacter* and *Serratia* showed the strongest hemolytic activity in vitro. Similarly, in the *Cx. pipiens* midgut, fluorescently labeled *Serratia* was shown to increase 700-fold after blood ingestion^34^. *Elizabethkingia* (Weeksellaceae) and *Stenotrophomonas* (Xanthomonadaceae) were increased after the blood meal in both isolines, in agreement with Telang and Skinner^32^, who observed *in Cx. quinquefasciatus* females, an increase in the relative abundance of *Elizabethkingia* after being fed pig blood. In particular, *E. anophelis*, previously found in the midgut of *Anopheles stephensi*, would play a role in blood metabolism^35^. In addition, *Stenotrophomonas* has been reported as an indicator species of *Ae. aegypti* gut microbiome after being fed chicken blood^36^.

Considering the fungal communities, the highest richness was observed in wt larvae; as we have suggested, this biota might be acquired during feeding and reduced after metamorphosis^16^. Studies on *Aedes* larvae have revealed that microorganism communities are more similar to the microbiota present in the breeding water than between mosquito populations from different breeding sites, suggesting that the environment is the dominant factor shaping the mycobiota present in the larvae^37, 38^.

Regarding fungal composition, the genus *Penicillium* was dominant in the sugar-fed mosquitoes, suggesting acquisition via sucrose solution. An inverse situation was observed in the blood-fed treated females, in which not only this OTU but also all fungal reads were extremely scarce. In engorged females, changes in the physiological state of the gut induce a selective environment for the bacterial microbiota^33, 39^, suggesting that gut conditions could possibly affect the fungal communities in the same way. As has been reported in *Ae. triseriatus* females, mycobiota abundance and composition change in the midgut as a response to blood intake^40^.

Multivariate analysis indicated that there were greater differences between both isolines for bacteria and lesser differences for fungal communities, suggesting that bacterial community structure in -tet isoline was affected by the antibiotic treatment (Fig. 3) due to the broad spectral action of tetracycline^41^. In mosquitoes^42^ and other insect hosts^43^, *Wolbachia* infection was found to affect the levels of reactive oxygen species (ROS), which are essential players in the immune system^42, 43^. Additionally, the presence of *Wolbachia* may influence the expression of phenoloxidase genes, antimicrobial effectors, and the load of bacteria and fungi^44^. Therefore, the effect of the antibiotic treatment in the initial mosquito generations, in addition to the reduction of *Wolbachia* influence in the restabilization of microbial communities, could be the cause of dominance/exclusion of certain bacterial genera.

The elimination of *Wolbachia* in mosquitoes using tetracycline has been identified as a successful method for its cure, possibly because of its bacteriostatic effect on bacterial populations, which would allow the reconstitution of the microbiota after suspending its application^41^. In this work, *Wolbachia* was detected in adults of the -tet isoline at a much lower abundance than in wt. Detection of genomic sequences by NGS has much higher sensitivity than endpoint PCR amplification^45^. Nevertheless, in previous work, CI was detected by crossing the two lines^15^.

In the case of fungi, the multivariate analysis did not produce a distinctive pattern between groups; therefore, no definitive role could be assigned to them in the differentiation between isolines. However, the high dissimilarity values of the larvae and blood-fed females of both isolines with the rest of the samples suggest that developmental stage and feeding are important factors in mycobiome differentiation.

Here, we demonstrated that richness, diversity, and microbial composition of *Cx. quinquefasciatus* depends on the characteristics of the initial microbiota, developmental stage, and female feeding. Furthermore, we suggest that the transmission and establishment of the found microbiota could be due to many factors, such as occupation of an ecological niche or competitive exclusion by dominant OTUs such as *Wolbachia*, *Thorsellia*, *Serratia* or *Penicillium*. Moreover, differential effects on vertical, transstage, and horizontal transmission, environmental influence, or changes in intestinal homeostasis by antibiotics or blood intake could be involved.

The role of the dominant microorganisms in the development and fitness of this mosquito should be further investigated, in order to analyze their potential for biotechnological applications aimed at managing insect populations or reducing their vectorial capacity.

### Supplementary Information

Supplementary Information.

## Data Availability

Data supporting the current investigation and the results reported in this article can be found at the NCBI repository (https://www.ncbi.nlm.nih.gov/). SRA database has been successfully processed with the BioProject name "16S Metagenome amplicon from *Culex quinquefasciatus*" (PRJNA925532, SUB12534211) with SRA accession numbers SRR23209995, SRR23209996, SRR23209999, SRR23210000, SRR23210001, SRR23209994, SRR23209997, and SRR23209998. Also, BioProject "Tetracycline-treated *Culex quinquefasciatus* ITS2 Fungi amplicon" (PRJNA925520, SUB12521670) with SRA accession numbers SRR23209969, SRR23209970, SRR23209971, SRR23209972, SRR23209966, SRR23209967, and SRR23209968. The SRA databases will be released once published.
