# Supplementary material for: Wolbachia dominance influences the Culex quinquefasciatus microbiota
Source: Sci Rep. 2023 Nov 3;13:18980. doi: 10.1038/s41598-023-46067-2 (PMC10624681; doi:10.1038/s41598-023-46067-2)
Supplement: Supplementary file 1 — Supplementary Information. [file 41598_2023_46067_MOESM1_ESM.zip › Suppl_Tables 1-2-5-6 and sfig legend.docx]

**Supplementary Files**

**Supplementary Table S1** Complete 16S V3-V4 ribosomal DNA region reads from mosquito stages: L: larvae, M: sucrose-fed male, SF: sucrose-fed females, BF: blood-fed females. Each library was constructed from groups of 20 individuals.

| **Sample ID** | **Total read bases** | | **Total reads** | | **GC(%)** | | **AT(%)** | | **Q20(%)** | | **Q30(%)** | |  |
| --- | --- | --- | --- | --- | --- | --- | --- | --- | --- | --- | --- | --- | --- |
| **L-tet_16S** | | 63,295,484 | | 210,284 | | 53.60 | | 46.40 | | 91.58 | | 82.29 | |
| **L_16S** | | 72,267,692 | | 240,092 | | 54.43 | | 45.57 | | 90.57 | | 80.73 | |
| **M-tet_16S** | | 67,555,236 | | 224,436 | | 52.48 | | 47.52 | | 91.85 | | 82.64 | |
| **M_16S** | | 58,342,830 | | 193,830 | | 50.31 | | 49.69 | | 92.36 | | 83.42 | |
| **F-tet_16S** | | 69,131,874 | | 229,674 | | 52.76 | | 47.24 | | 92.83 | | 84.23 | |
| **F_16S** | | 70,318,416 | | 233,616 | | 48.35 | | 51.65 | | 93.01 | | 84.48 | |
| **BF-tet_16S** | | 63,493,542 | | 210,942 | | 52.94 | | 47.06 | | 92.49 | | 83.81 | |
| **BF_16S** | | 75,853,204 | | 252,004 | | 47.84 | | 52.16 | | 93.63 | | 85.57 | |
| **Total** | | 540,258,278 | | 1,794,878 | |  | |  | |  | |  | |

Sample ID: Sample name.

Total read bases: Total number of bases sequenced.

Total reads: Total number of reads. For Illumina paired-end sequencing, this value refers to the sum of read 1 and read 2.

GC(%): GC content.

AT(%): AT content.

Q20(%): Ratio of bases that have a phred quality score of over 20.

Q30(%): Ratio of bases that have a phred quality score of over 30.

**Supplementary Table S2** Complete ITS2 ribosomal DNA region reads from mosquito stages: L: larvae, M: sucrose-fed male, SF: sucrose-fed females, BF: blood-fed females. Each library was constructed from groups of 20 individuals.

| **Sample ID** | **Total read bases** | **Total reads** | **GC(%)** | **AT(%)** | **Q20(%)** | **Q30(%)** |
| --- | --- | --- | --- | --- | --- | --- |
| **L Set 1** | 45,681,566 | 151,766 | 54.62 | 45.38 | 85.91 | 74.82 |
| **M Set 1** | 42,138,796 | 139,996 | 54.65 | 45.35 | 78.31 | 66.98 |
| **SF Set 1** | 48,486,886 | 161,086 | 54.55 | 45.45 | 78.47 | 67.07 |
| **BF Set 1** | 68,732,748 | 228,348 | 54.11 | 45.89 | 87.99 | 77.95 |
| **L Set 2** | 84,491,302 | 280,702 | 53.64 | 46.36 | 83.18 | 72.95 |
| **M Set 2** | 84,890,428 | 282,028 | 54.84 | 45.16 | 83.78 | 73.13 |
| **SF Set 2** | 9,895,032 | 265,432 | 54.72 | 45.28 | 83.6 | 72.91 |
| **Total** | 384,316,758 | 1509,358 |  |  |  |  |

Sample ID: Sample name.

Total read bases: Total number of bases sequenced.

Total reads: Total number of reads. For Illumina paired-end sequencing, this value refers to the sum of read 1 and read 2.

GC(%): GC content.

AT(%): AT content.

Q20(%): Ratio of bases that have phred quality score of over 20.

Q30(%): Ratio of bases that have phred quality score of over 30.

**Supplementary Table S3** Bacterial OTUs (xslx file).

**Supplementary Table S4** Fungal OTUs (xslx file).

**Supplementary Table S5** Bray-Curtis distance at the bacterial OTUs level among all mosquito group.

| ***16S*** | **L** | **L-tet** | **M** | **M-tet** | **SF** | **SF-tet** | **BF** | **BF-tet** |
| --- | --- | --- | --- | --- | --- | --- | --- | --- |
| **L** |  |  |  |  |  |  |  |  |
| **L-tet** | 0.66 |  |  |  |  |  |  |  |
| **M** | 0.66 | 0.65 |  |  |  |  |  |  |
| **M-tet** | 0.70 | 0.28 | 0.63 |  |  |  |  |  |
| **SF** | **0.88** | **0.97** | 0.47 | **0.94** |  |  |  |  |
| **SF-tet** | 0.68 | 0.59 | 0.52 | 0.56 | **0.83** |  |  |  |
| **BF** | **0.88** | **0.96** | 0.52 | **0.92** | 0.14 | **0.82** |  |  |
| **BF-tet** | 0.73 | 0.47 | 0.60 | 0.46 | **0.91** | 0.33 | **0.89** |  |

**Supplementary Table S6** Bray-Curtis distance at the fungal OTUs level among all mosquito group.

| ***ITS*** | **L** | **L-tet** | **M** | **M-tet** | **SF** | **SF-tet** | **BF** | **BF-tet** |
| --- | --- | --- | --- | --- | --- | --- | --- | --- |
| **L** |  |  |  |  |  |  |  |  |
| **L-tet** | 0.76 |  |  |  |  |  |  |  |
| **M** | 0.43 | **0.96** |  |  |  |  |  |  |
| **M-tet** | 0.49 | **0.97** | 0.37 |  |  |  |  |  |
| **SF** | 0.69 | **0.96** | 0.4 | 0.66 |  |  |  |  |
| **SF-tet** | **0.89** | **0.96** | 0.72 | **0.81** | 0.58 |  |  |  |
| **BF** | **1** | **1** | **1** | **1** | **0.99** | **0.98** |  |  |
| **BF-tet** | **1** | **1** | **0.99** | **1** | **0.99** | **0.98** | **1** |  |

**Supplementary Figure S1** Bacterial rarefaction curves described for observed OTUs metric among all mosquito groups: L: larvae, M: sucrose-fed male, SF: sucrose-fed females, BF: blood-fed females; in lines with (-tet) or without tetracycline treatment. The rarefied number of operational taxonomic units (OTUs, with 97% sequence similarity cut-off value) is plotted against the number of reads sampled.

**Supplementary Figure S2** Fungal rarefaction curves described for observed OTUs metric among all mosquito groups: L: larvae, M: sucrose-fed male, SF: sucrose-fed females, BF: blood-fed females; in lines with (-tet) or without tetracycline treatment. The rarefied number of operational taxonomic units (OTUs, with 97% sequence similarity cut-off value) is plotted against the number of reads sampled.
