# Supplementary figures and images for: Wolbachia dominance influences the Culex quinquefasciatus microbiota
Source: Sci Rep. 2023 Nov 3;13:18980. doi: 10.1038/s41598-023-46067-2 (PMC10624681; doi:10.1038/s41598-023-46067-2)

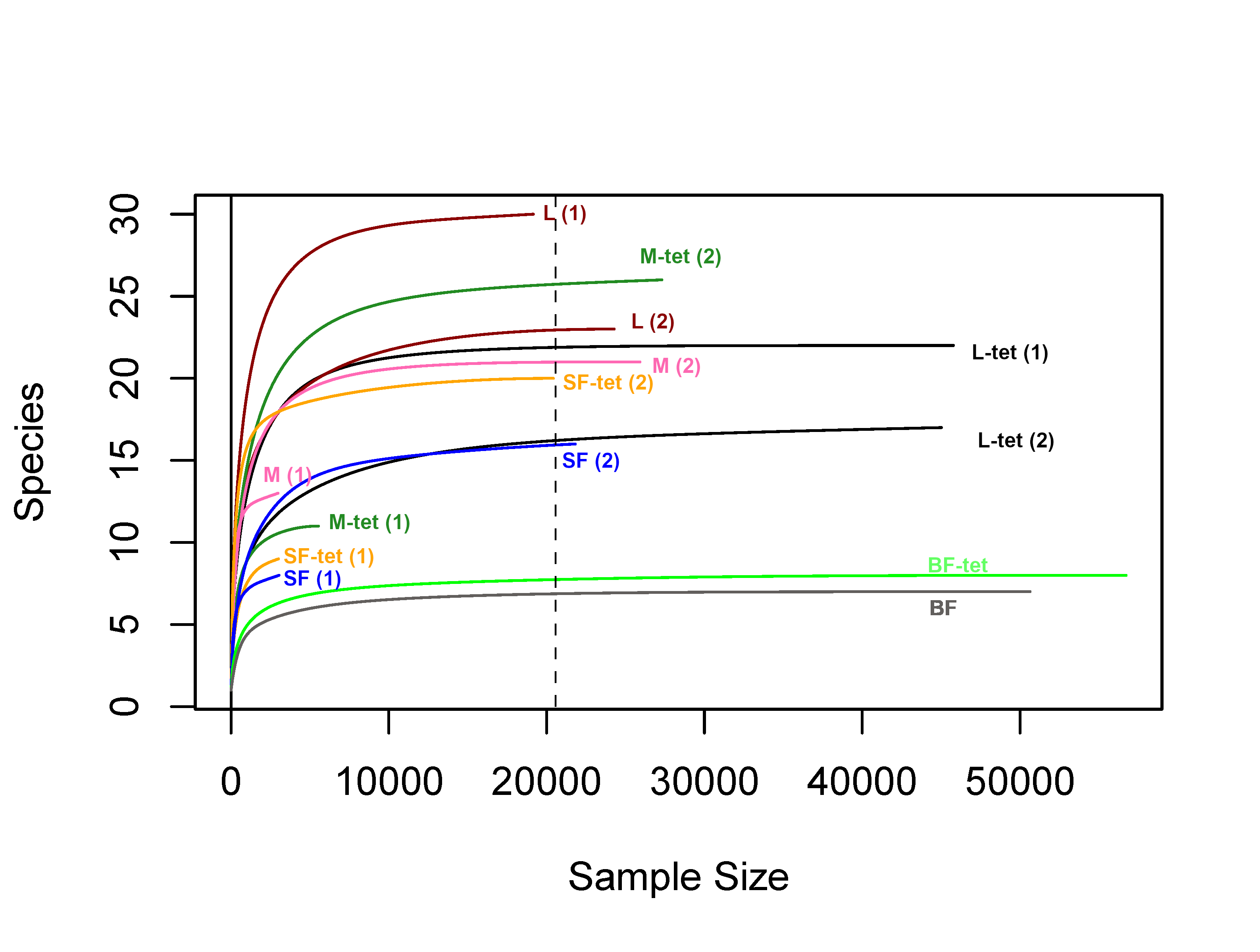

Supplement: Supplementary file 1 — Supplementary Information. [file 41598_2023_46067_MOESM1_ESM.zip › Fig S2.tiff]

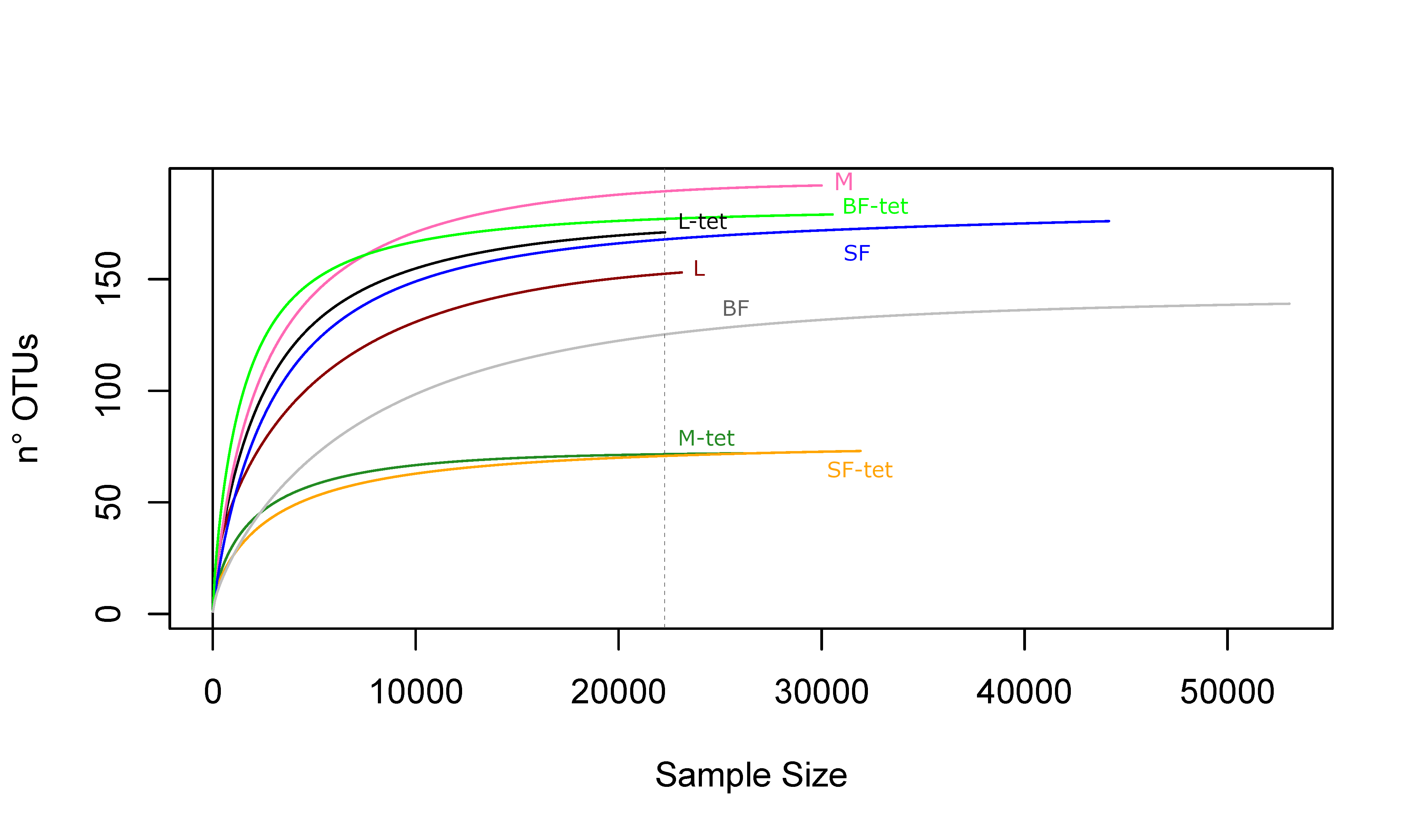

Supplement: Supplementary file 1 — Supplementary Information. [file 41598_2023_46067_MOESM1_ESM.zip › FigS1.tiff]
